# Clinical Characteristics, Cardiometabolic Risk Burden and Therapeutic Patterns in Patients Attending Cardiology Clinics in Spain: The RIESCAR Study

**DOI:** 10.3390/jcm15207916

**Published:** 2026-10-14

**Authors:** Juan Cosín-Sales, Raquel Campuzano, Angel Cequier, Pablo Díez-Villanueva, Albina Aldomà-Balasch, Vicente Ignacio Arrarte-Esteban, Eduardo Sebastián López-Sánchez, Casimiro Gómez-Pajuelo, Leticia Fernández-Friera, José Miguel Chopo-Alcubilla, Javier Muñiz, Juan José Gómez-Doblas

**Affiliations:** 1 Servicio de Cardiología, Hospital Universitario Arnau de Vilanova, 46015 Valencia, Spain; 2 Facultad de Medicina, Universidad CEU Cardenal Herrera, 46115 Valencia, Spain; 3 Servicio de Cardiología, Hospital Universitario Fundación Alcorcón, 28922 Madrid, Spain; 4 Servicio de Cardiología, Hospital Universitario de Bellvitge, Instituto de Investigación Biomédica de Bellvitge (IDIBELL), 08907 L’Hospitalet de Llobregat, Spain; 5 Servicio de Cardiología, Hospital Universitario de La Princesa, IIS-IP, 28006 Madrid, Spain; 6 Servicio de Cardiología, Hospital Comarcal de Móra d’Ebre, 43740 Tarragona, Spain; 7 Institut de Recerca Biomèdica Catalunya Sud (IRBCatSud), 43500 Tortosa, Spain; 8 Servicio de Cardiología, Hospital General Universitario Dr. Balmis, 03010 Alicante, Spain; 9 Servicio de Cardiología, Clínica Cardiológica Vera, 04638, Almería, Spain; 10 Servicio de Cardiología, Medicentro Leganés, 28912 Madrid, Spain; 11 HM CIEC Madrid (Centro Integral de Enfermedades Cardiovasculares), Hospital Universitario HM Montepríncipe, HM Hospitales, Facultad HM de Ciencias de la Salud, Universidad Camilo José Cela, Instituto de Investigación Sanitaria HM Hospitales, 28660 Madrid, Spain; 12 Network Biomedical Research Centre for Cardiovascular Diseases (CIBER-CV), Carlos III Health Institute, 28029 Madrid, Spain; javmu@udc.es (J.M.); jjgomezdoblas@secardiologia.es (J.J.G.-D.); 13 Servicio de Cardiología, Clínica de Cardiología Dr. Chopo Alcubilla, 50004 Zaragoza, Spain; 14 Grupo de Investigación Cardiovascular, Departamento de Ciencias de la Salud, 15006 Coruña, Spain; 15 Instituto de Investigación Biomédica de A Coruña (INIBIC), Universidade da Coruña, 15006 Coruña, Spain; 16 Institute of Biomedical Research of Málaga and Nanomedicine Platform (IBIMA-Plataforma BIONAND), Andalusia Technology Park (PTA), Severo Ochoa 35, 29590 Málaga, Spain; 17 Department of Cardiology and Cardiovascular Surgery-Heart Area, Virgen de la Victoria University Hospital, 29010 Málaga, Spain

**Keywords:** cardiometabolic disease, central obesity, outpatient cardiology, preventive cardiology, LDL cholesterol, hypertension

## Abstract

**Background/Objectives**: Contemporary data on patients attending cardiology outpatient clinics in Spain are scarce. We aimed to characterise cardiovascular conditions, cardiometabolic burden, risk-factor control and treatment patterns. **Methods**: RIESCAR is a national, multicentre, observational study. This analysis included 2808 adults evaluated in 50 general cardiology outpatient clinics. **Results**: Hypertension was present in 60.3%, dyslipidaemia in 58.9%, type 2 diabetes mellitus in 24.7%, obesity in 32.4%, and central obesity in 48.7%. Established cardiovascular disease was documented in 59.7%, with coronary artery disease (30.0%), atrial fibrillation (18.2%) and heart failure (11.7%) being the most frequent conditions. Among hypertensive patients, 42.6% had blood pressure ≥ 140/90 mmHg. In patients with established atherosclerotic cardiovascular disease (ASCVD), 37.2% achieved LDL-C < 55 mg/dL. Patients received a high number of cardiovascular drugs (mean 3.9). Among patients with a potential clinical indication for contemporary cardiometabolic therapies, SGLT2 inhibitors and GLP-1 receptor agonists were used in 35.4% and 8.6%, respectively. **Conclusions**: Patients attending cardiology clinics in Spain have a high cardiometabolic burden, with important gaps in blood-pressure and LDL-C target attainment. Use of contemporary cardiometabolic therapies remains variable, highlighting opportunities to strengthen cardiovascular prevention in routine practice.

## 1. Introduction

Cardiovascular disease (CVD) remains the leading cause of morbidity and mortality worldwide and continues to impose a major clinical and economic burden in Spain [1]. Although advances in acute cardiovascular care have improved survival after acute events, this has resulted in a growing population of patients living longer with chronic CVD [2]. Consequently, some cardiology outpatient clinics have increasingly evolved into long-term follow-up settings for complex, multimorbid patients rather than purely diagnostic or episodic care environments.

In parallel, improvements in electronic health care systems have strengthened coordination with primary care and markedly increased the use of electronic consultations in recent years [3]. These organisational changes are likely to have modified the profile of patients managed in cardiology outpatient clinics; however, contemporary data characterising this population in Spain remain limited.

Most patients who experience a cardiovascular event—such as coronary artery disease (CAD) or heart failure (HF)—are evaluated by a cardiologist at some point during their clinical course. In addition, many patients are referred to cardiology outpatient clinics for suspected cardiovascular disease, hypertension, palpitations, or cardiovascular risk assessment. A substantial proportion of these individuals, despite lacking established CVD, present a high-risk profile characterised by smoking, hypertension, diabetes, obesity, or advanced age. In this context, the cardiology consultation represents a critical opportunity to initiate or reinforce preventive strategies. Therefore, the goal of cardiology outpatient practice should extend beyond treating established disease to actively and opportunistically preventing its onset.

Alongside population ageing, one of the most profound epidemiological changes affecting cardiovascular health in recent decades has been the rise in cardiometabolic disease. Obesity—particularly central obesity—has emerged as a powerful determinant of cardiovascular risk across the clinical spectrum [4]. While body mass index (BMI) offers only a partial approximation of adiposity, waist circumference, as a marker of visceral fat accumulation, is strongly linked to insulin resistance, inflammation and HF with preserved ejection fraction [5].

At the same time, therapeutic options for cardiometabolic disease have expanded. SGLT2 inhibitors and GLP-1 receptor agonists provide significant cardiovascular and renal benefits, promote weight loss, reduce visceral adiposity, and improve metabolic control, representing a paradigm shift in preventive cardiology [6,7,8,9,10,11,12,13]. However, the extent to which these therapies have been incorporated into cardiology outpatient practice in Spain remains uncertain. Existing registries in Spain tend to focus on specific diseases—such as CAD [14], atrial fibrillation [15], or HF [16]—rather than offering a comprehensive picture of the broad population attending cardiology outpatient clinics.

The RIESCAR study was designed to fill this gap by providing an updated, detailed, and real-world characterisation of patients evaluated in cardiology outpatient settings in Spain, with the broader objective of improving the understanding of their cardiometabolic burden, clinical course, and long-term outcomes through a mixed cross-sectional and longitudinal design. The aim of the present manuscript is to describe the cross-sectional (baseline) phase of the RIESCAR study, providing a comprehensive characterisation of patients attending cardiology clinics, with particular emphasis on central obesity, multimorbidity, metabolic dysfunction, suboptimal lipid control, and the limited penetration of modern cardiometabolic therapies.

## 2. Materials and Methods

### 2.1. Study Design and Setting

RIESCAR is a national, multicentre, observational study with a mixed cross-sectional and longitudinal design, conducted in cardiology outpatient clinics across Spain. This manuscript reports the cross-sectional phase of the study and includes patients evaluated in 50 general cardiology outpatient clinics, including tertiary hospitals, regional hospitals, community centres and private cardiology practices. Baseline recruitment was conducted from 5 September 2024 to 15 May 2025, and the number of participants recruited at each centre is provided in Supplementary Table S1.

Participating centres were invited centrally by the Research Agency of the Spanish Society of Cardiology (AISEC), with the aim of achieving a broad and geographically balanced representation of cardiology outpatient practice across Spain. Participating clinics were required to provide general cardiology care and to avoid highly specialised or disease-specific settings, in order to reflect routine clinical practice. Participation was voluntary and the final sample was not probability-based.

Investigators at each participating centre were specifically instructed to recruit eligible patients consecutively and agreed to follow this procedure throughout the inclusion period. However, no central screening log or independent monitoring system was available to verify complete adherence to consecutive recruitment at each site. The study was promoted and coordinated by AISEC, which oversaw the participation of centres throughout Spain.

### 2.2. Participants and Ethics

Adult patients aged 18 to 80 years attending scheduled cardiology outpatient visits for any reason—including cardiovascular risk assessment, evaluation of symptoms, routine follow-up, or long-term disease management—were eligible for inclusion. No exclusion criteria were applied with regard to comorbidities, disease severity, or ongoing pharmacological treatments. This approach was intended to provide a broad characterisation of contemporary general cardiology outpatient practice rather than a nationally representative population estimate.

This study was conducted according to the guidelines of the Declaration of Helsinki and approved by the Ethics Committee of the Hospital Arnau de Vilanova (act number 12_2023) in Valencia, Spain (code: EOm 23_2023, date: 21 December 2023). Signed informed consent was obtained from all subjects. The study complies with current data protection regulations under Spanish law.

During preparation of this manuscript, the authors used OpenAI ChatGPT (GPT-5.6 Sol; OpenAI, San Francisco, CA, USA; accessed on 18 September 2026) to assist with language editing, reference formatting and development of the graphical abstract. All AI-assisted output was critically reviewed, edited and verified by the authors. No generative AI tool was used to generate, analyse or interpret the study data.

### 2.3. Data Collection and Variable Definitions

Data were collected using an electronic standardised case report form. Sociodemographic variables included age, sex, educational level, living situation, and employment status. Cardiovascular risk factors recorded were hypertension, dyslipidaemia, diabetes mellitus, smoking status, and obesity. Alcohol consumption and recreational drug use were also documented.

Information on established CVD was collected, including CAD, myocardial infarction subtype, prior coronary revascularisation, atrial fibrillation, cardiomyopathies, valvular heart disease, HF phenotype, aortic disease, congenital heart disease, and the presence of implanted cardiac devices. Pacemaker status was recorded as ventricular or dual-chamber, while ICD/cardiac resynchronisation therapy (CRT) was recorded as ICD alone, CRT alone, or both. Recorded non-cardiovascular comorbidities included chronic kidney disease, dialysis use, liver disease, chronic obstructive pulmonary disease, obstructive sleep apnoea, cancer history and type, thyroid dysfunction, and peripheral arterial disease. History of myocarditis, acute or prior pericarditis, and infective endocarditis was not systematically collected; only chronic pericardial disease was specifically recorded.

Lifestyle-related variables were collected at the baseline visit. Physical activity was assessed using the short form of the International Physical Activity Questionnaire (IPAQ-7).

Anthropometric measurements included weight, height, BMI, and waist circumference, measured at the midpoint between the costal margin and the iliac crest. Vital signs recorded at the visit included blood pressure and heart rate. No study-specific standardised blood-pressure measurement protocol or repeated study measurement was mandated; blood pressure was recorded according to routine clinical practice at each participating centre.

Complementary cardiovascular testing included electrocardiographic and echocardiographic data, such as cardiac rhythm, conduction abnormalities, left ventricular ejection fraction, structural abnormalities, and valvular lesions.

Laboratory parameters collected included lipid profile, lipoprotein(a), renal function, urinary protein concentration, high-sensitivity C-reactive protein (hs-CRP), and glycated haemoglobin (HbA1c). Tests were recorded when available from routine clinical care and were not mandated by the protocol.

Pharmacological treatment was comprehensively recorded and categorised by therapeutic class, including antiplatelet agents, statins, ezetimibe, PCSK9 inhibitors, angiotensin-converting enzyme inhibitors/angiotensin receptor blockers, beta-blockers, mineralocorticoid receptor antagonists, sodium–glucose cotransporter 2 inhibitors, GLP-1 receptor agonists, oral hypoglycaemic agents, and insulin. Total cardiovascular therapeutic burden was defined as the number of cardiovascular drugs prescribed per patient.

Within the overall RIESCAR study design, a subset of patients identified at the baseline visit with established atherosclerotic cardiovascular disease and/or type 2 diabetes mellitus were invited to participate in a longitudinal follow-up, with planned assessments at one and three years; however, longitudinal data are not analysed in the present manuscript.

Recent past smoker was considered if <1 year since cessation, and past smoker if >1 year since cessation. Central obesity was defined as waist circumference > 102 cm in men and >88 cm in women. Obesity was defined as BMI ≥ 30 kg/m^2^. In addition, patients with BMI > 27 kg/m^2^ were specifically identified in the analyses, as this threshold was used as an inclusion criterion in recent cardiovascular outcome trials with GLP-1 receptor agonists, such as the SELECT trial [12], and defines a population potentially eligible to benefit from these therapies.

Established CVD included history of CAD, myocardial infarction, HF, atrial fibrillation, significant valvular disease, cardiomyopathies, congenital heart disease, aortic pathology, or cardiac device implantation. This broad composite was used to characterise the overall cardiology case mix and was not intended to represent atherosclerotic disease. For analyses specifically related to atherosclerotic risk and lipid-lowering therapy, established atherosclerotic cardiovascular disease (ASCVD) was defined as ischaemic heart disease, ischaemic stroke, or peripheral arterial disease. The proportion of these patients with LDL-C < 55 mg/dL was calculated using the ESC/EAS recommended threshold [17].

Chronic kidney disease was considered if glomerular filtration rate < 60 mL/min, or in the presence of a previous diagnosis or dialysis use. Liver disease was considered in the presence of a previous diagnosis or elevation of liver enzymes > 3 times the upper limit of normal on more than one occasion. When available, urinary protein concentration (mg/dL) was recorded as reported in the patients’ clinical records; urinary albumin-to-creatinine ratio was not systematically collected.

### 2.4. Statistical Analysis

Continuous quantitative variables were expressed as mean ± SD or median and interquartile range, depending on their distribution, while categorical variables were expressed as frequencies and percentages. Analyses were descriptive and exploratory in nature. A focused secondary comparison of key baseline characteristics between patients with and without established CVD was performed using *t*-tests or chi-square tests as appropriate and is presented in Supplementary Table S2. These comparisons were not intended to estimate independent associations. Although the patient-level centre identifier was available, no centre-level inferential model was prespecified; therefore, these exploratory *p* values were not adjusted for clustering by centre and should be interpreted accordingly. Population proportions were estimated using point estimates with 95% confidence intervals. Missing data were not imputed. Denominators are reported for variables with incomplete ascertainment, and the possibility of indication-related selection should be considered for laboratory tests performed according to clinical indication.

## 3. Results

A total of 2808 patients from 50 cardiology outpatient clinics were included between 5 September 2024 and 15 May 2025. Recruitment by centre ranged from 10 to 110 participants (Supplementary Table S1). The study population had a mean age of 63 years and was predominantly male, with women representing 36.7%; most patients were retired or living with a partner, reflecting the demographic profile of a chronic cardiovascular outpatient population (Table 1). A focused comparison of key baseline characteristics between patients with and without established CVD is presented in Supplementary Table S2.

Cardiometabolic risk factors were highly prevalent. Hypertension and dyslipidaemia affected the majority of participants, and one in four patients had diabetes mellitus, mostly type 2. Smoking history remained relevant, as more than a third were former smokers and a smaller but meaningful proportion continued to smoke. Alcohol and recreational drug abuse were infrequently documented. The distribution of documented alcohol/recreational drug abuse according to established CVD status is presented in Supplementary Table S3. With regard to physical activity, fewer than half of the participants reported performing at least 150 min of aerobic exercise per week (Table 1).

The anthropometric profile revealed a substantial burden of excess adiposity. A BMI > 27 kg/m^2^ was present in 55.7% of participants, while obesity (BMI ≥ 30 kg/m^2^) was present in 907/2803 patients (32.4%). Obesity was more common in patients with established CVD (34.1%) than in those without established CVD (29.8%; *p* = 0.016). Waist circumference averaged approximately 98 cm, and 48.7% of the cohort met criteria for central obesity (Table 2). In addition, 571/2774 participants with both BMI and waist-circumference data available (20.6%) had central obesity despite BMI < 30 kg/m^2^.

Vital signs (Table 2) showed mean systolic and diastolic blood pressure values of 130.9 ± 18.7 and 76.0 ± 10.8 mmHg, respectively. Among patients with diagnosed hypertension, 57.4% had a clinic blood pressure < 140/90 mmHg at the study visit. Heart rate was significantly higher in patients with atrial fibrillation on the baseline ECG than in those in sinus rhythm (78.14 ± 16.31 vs. 69.42 ± 11.85 bpm, *p* < 0.01).

**Figure 1:**
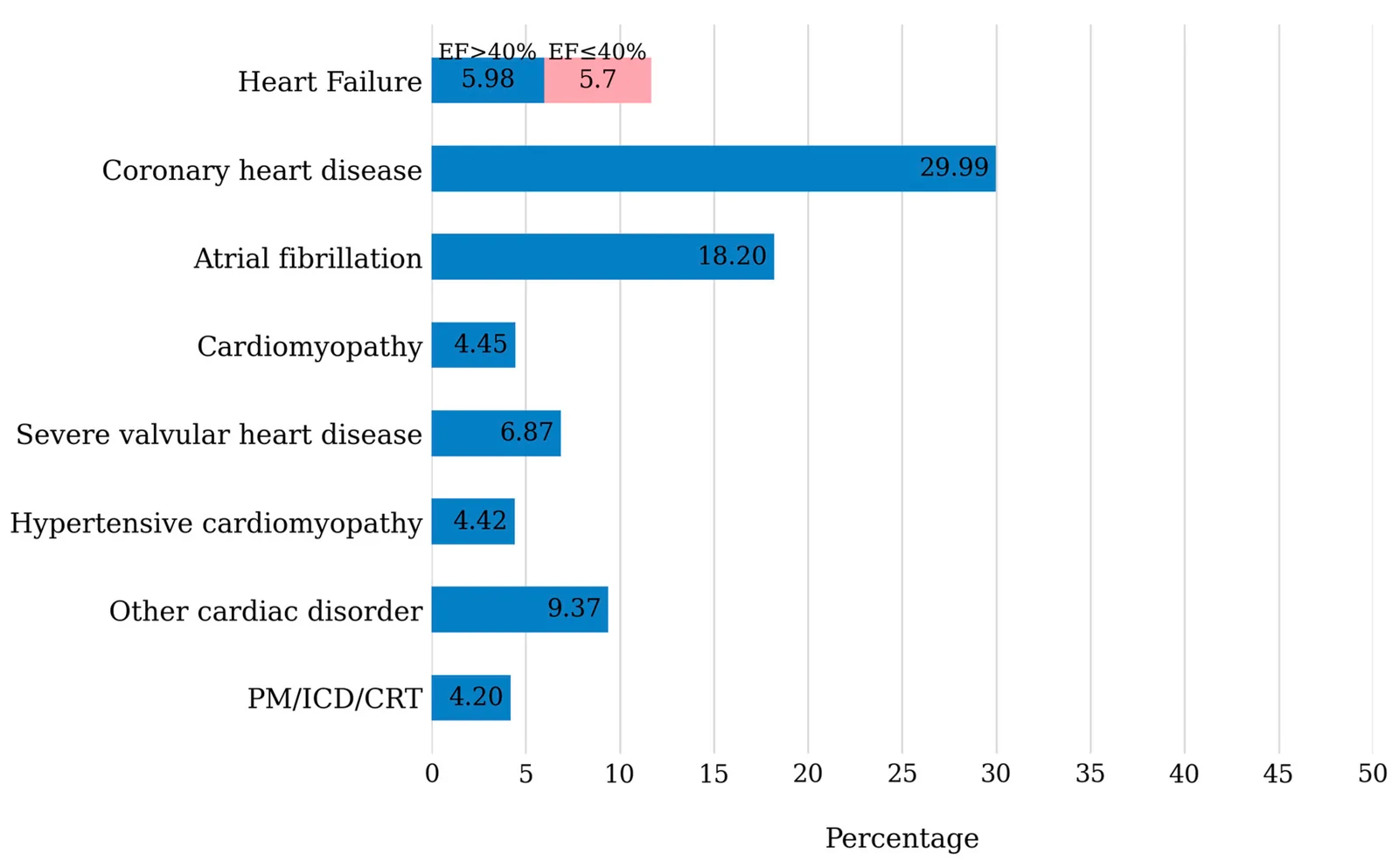
Distribution of cardiovascular diagnoses in the RIESCAR cohort. Percentages are calculated using the entire study population (n = 2808) as a denominator; diagnostic categories are not mutually exclusive. Heart failure is shown by LVEF category (>40%: 6.0%; ≤40%: 5.7%; total approximately 11.7%). PM, pacemaker; ICD, implantable cardioverter–defibrillator; CRT, cardiac resynchronisation therapy; EF, ejection fraction.

As expected in a cardiology outpatient setting, established CVD was common. However, 40.3% of patients (1131/2808) had no documented history of CVD. Among patients with established disease, CAD was the most prevalent diagnosis (Figure 1). A large proportion of these patients had experienced at least one prior myocardial infarction, most frequently non-ST-segment elevation myocardial infarction (46.79%). Previous coronary revascularisation was common (83.61%), with percutaneous coronary intervention being the predominant modality (69.83%). Atrial fibrillation was documented in the medical history of 511 patients (18.2%), and HF was present in approximately 11.7% of the cohort. Cardiomyopathies, significant valvular heart disease, hypertensive heart disease, congenital heart disease, and implanted devices were also represented (Figure 1). Pacemakers were present in 2.28% of patients and ICD and/or CRT devices in 2.21%. Additional recorded conditions included chronic pericardial disease (0.25%), congenital heart disease (1.32%), moderate or severe pulmonary hypertension (1.07%), and other structural heart diseases (7.09%).

Non-cardiovascular comorbidities were frequent and added substantially to the overall complexity of the cohort. Obstructive sleep apnoea was present in 10.2% of patients, chronic kidney disease in 9.9%, cancer history in 8.4%, hypothyroidism in 8.2%, and chronic obstructive pulmonary disease in 6.5%; prior ischaemic stroke or transient ischaemic attack was documented in 4.5%. Peripheral, aortic, or carotid arterial disease was recorded in 5.8% of the cohort. This multimorbidity profile likely increases therapeutic complexity and may limit treatment options, particularly in patients requiring multiple concomitant medications.

Electrocardiographic and echocardiographic findings were consistent with these clinical profiles. Sinus rhythm predominated on the baseline ECG (88.7%), whereas atrial fibrillation was present on that single ECG in 267/2805 patients (9.5%). This differs from the 18.2% prevalence of clinically documented atrial fibrillation because the latter reflects a prior diagnosis irrespective of rhythm at the study visit: among 511 patients with a history of atrial fibrillation, 220 were in atrial fibrillation, 271 in sinus rhythm, 18 in a paced rhythm, and 2 in another rhythm at baseline. Conduction abnormalities were identified in 14.8%, with right and left bundle branch block occurring in approximately 6.5% each. Echocardiography showed a largely preserved systolic profile, with a mean LVEF of 60.2 ± 9.1%, while left ventricular hypertrophy was observed in 29.3%.

Laboratory findings (Table 3) highlighted important aspects of preventive cardiology. Mean LDL-C was 93 mg/dL. Among patients with established ASCVD, only 365/981 (37.2%) had LDL-C < 55 mg/dL. Availability varied substantially across laboratory variables: LDL-C was available in 97.4% of the cohort and HbA1c in 65.1%, whereas Lp(a), hs-CRP, and urinary protein concentration were available in only 8.8%, 18.1%, and 19.2%, respectively. These findings reflect the limited use of these additional risk-marker assessments in routine cardiology outpatient practice.

**Figure 2:**
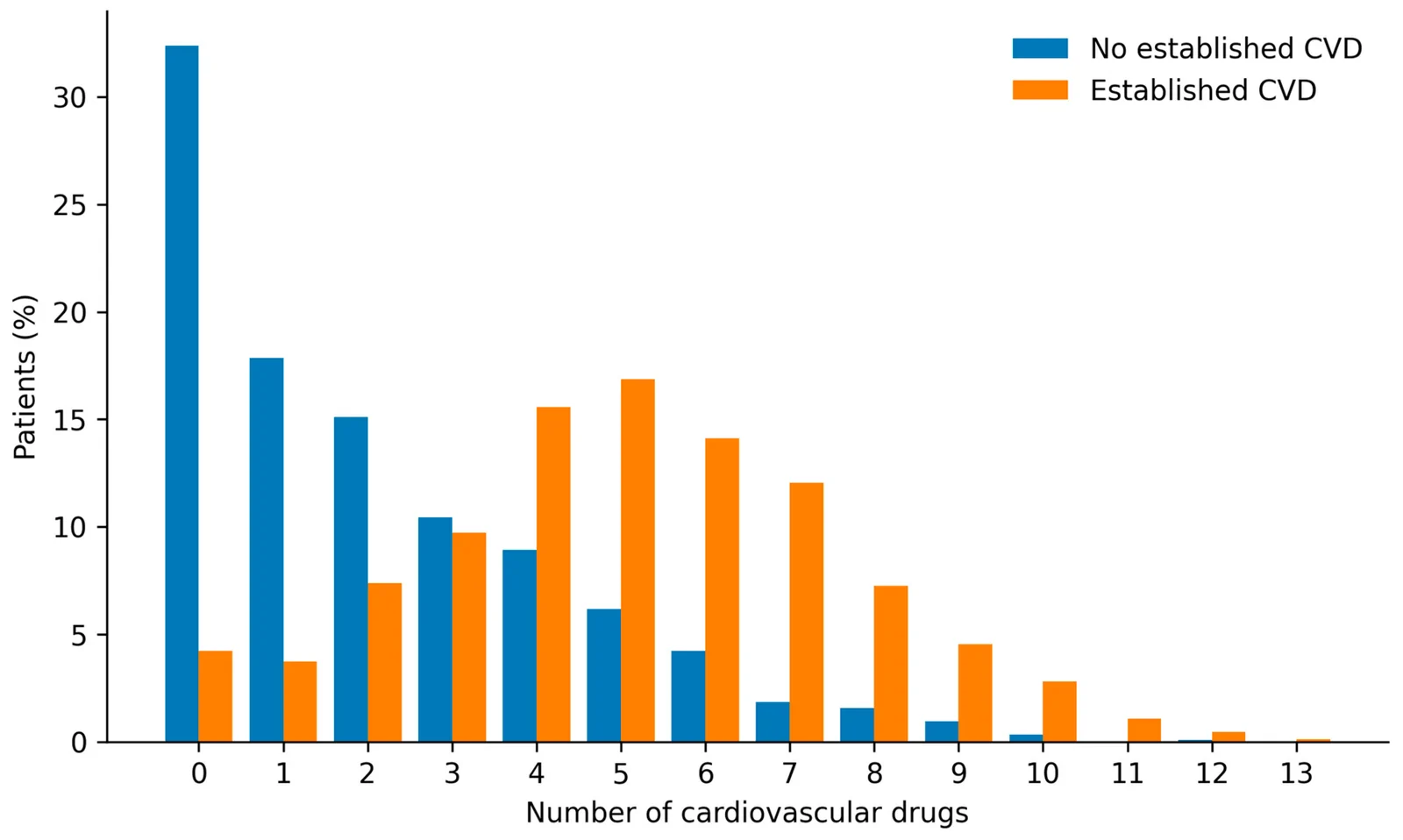
Distribution of the number of cardiovascular drugs per patient according to the presence or absence of established cardiovascular disease (CVD).

Medication patterns reflected the complex therapeutic needs of this population. Patients were taking a mean of 3.89 ± 2.81 cardiovascular drugs, rising to 5.10 ± 2.50 in patients with established CVD versus 2.11 ± 2.23 in those without CVD (*p* < 0.001) (Figure 2). In the overall cohort, 59.9% received statins, 28.1% ezetimibe, 1.1% PCSK9 monoclonal antibodies, 0.4% inclisiran, 3.0% bempedoic acid, 24.0% SGLT2 inhibitors, and 5.8% GLP-1 receptor agonists (Table 4). Indication-related subgroup analyses provided additional context (Supplementary Tables S4 and S5). Among 990 patients with ASCVD, 91.7% received a statin, 60.0% ezetimibe, 7.7% bempedoic acid, 2.8% a PCSK9 monoclonal antibody, and 1.0% inclisiran; 58.6% received statin plus ezetimibe. SGLT2 inhibitors were used in 61.8% of patients with diabetes mellitus, 73.8% with HF, 48.8% with chronic kidney disease, 30.7% with obesity and 37.0% with ASCVD. GLP-1 receptor agonists were used in 18.3%, 7.6%, 9.0%, 13.5% and 8.8% of these respective subgroups. Among 511 patients with a history of atrial fibrillation, 447 (87.5%) received anticoagulation: 96 (21.5%) a vitamin K antagonist, 348 (77.9%) a direct oral anticoagulant, and 3 (0.7%) another anticoagulant. Thromboembolic and bleeding-risk scores were not collected; therefore, the proportion of anticoagulation among patients with a formal guideline indication could not be determined.

## 4. Discussion

The RIESCAR study provides valuable insights into the current landscape of cardiology outpatient care in Spain and highlights several findings with important clinical implications. One of the main findings of the study is that, contrary to what might be expected, cardiology outpatient clinics do not exclusively manage patients with established CVD. Indeed, more than 40% of patients had no prior documented history of CVD. This observation underscores a substantial and often underrecognised opportunity for cardiologists to play a central role in primary prevention by implementing and reinforcing evidence-based preventive strategies in a large proportion of patients.

An additional relevant finding was the underrepresentation of women, who accounted for only 36% of the study population. This observation is consistent with previous reports from cardiology registries [18] and may reflect a combination of referral patterns, differences in disease presentation, and persistent sex-related disparities in access to specialised cardiovascular care. When focusing on patients with established CVD, CAD was the most prevalent condition managed in cardiology outpatient clinics. Most of these individuals had previously undergone coronary revascularisation, reflecting both the high burden of advanced CAD and the need for sustained secondary prevention. Atrial fibrillation and HF were also commonly managed in outpatient cardiology practice and contributed substantially to the overall morbidity burden of the population. In contrast, severe valvular heart disease and cardiomyopathies accounted for a smaller proportion of outpatient visits, indicating that while these conditions are clinically relevant, they represent a more limited share of routine cardiology outpatient care.

Another striking finding of the RIESCAR study is the extraordinarily high prevalence of central obesity. This observation has major pathophysiological and clinical implications. Waist circumference, more so than BMI, reflects visceral adiposity and its downstream consequences, including insulin resistance, systemic inflammation, autonomic dysfunction, and increased susceptibility to both atherosclerosis and cardiac arrhythmias [4]. In routine clinical practice, however, waist circumference is still inconsistently measured. The present findings strongly suggest that failure to routinely assess central obesity may lead to a substantial underestimation of cardiometabolic risk in a large proportion of patients attending cardiology clinics. Systematic incorporation of waist-circumference measurement into outpatient assessment could facilitate earlier identification of high-risk phenotypes and enable more targeted preventive interventions [19].

In this context, the limited use of GLP-1 receptor agonists is particularly noteworthy. Despite robust evidence demonstrating cardiovascular benefit and weight reduction, the penetration of GLP-1 receptor agonists in cardiology outpatient practice remains very low. In RIESCAR, GLP-1 receptor agonists were prescribed to 18.3% of patients with diabetes mellitus and 13.5% of those with obesity. Several factors may contribute to this gap. Reimbursement restrictions for patients with obesity without poorly controlled diabetes may represent an important barrier, despite compelling results from the SELECT trial showing cardiovascular benefit in patients with obesity without diabetes [12]. In addition, traditional speciality boundaries may play a role, as GLP-1 receptor agonists are often perceived as therapies primarily managed by endocrinologists. Until recently, cardiology guidelines have also provided limited guidance regarding the use of these agents in cardiovascular prevention. The RIESCAR data suggest that broader adoption of GLP-1 receptor agonists within cardiology practice could substantially improve outcomes by simultaneously addressing metabolic dysfunction and atherosclerotic risk.

The limited assessment of renal risk markers deserves particular attention. Urinary protein concentration was available in only 19.2% of participants, and urinary albumin-to-creatinine ratio (UACR) was not systematically collected. This is notable because current guidelines recommend UACR as part of the routine assessment of patients with hypertension and at least annually in all patients with type 2 diabetes [20,21]. Given that 60.3% of the cohort had hypertension and 24.7% had type 2 diabetes, these findings suggest an important opportunity to improve systematic albuminuria assessment in cardiology outpatient practice. Earlier identification of albuminuria may refine cardiovascular and renal risk stratification and inform evidence-based cardiorenal therapy. The low availability of Lp(a) and hs-CRP similarly indicates that these additional risk markers are not routinely assessed in contemporary cardiology outpatient practice.

Another important finding is the persistent gap between guideline recommendations for lipid management and real-world lipid control. Earlier Spanish studies in very-high-risk populations reported low achievement of guideline-recommended LDL-C targets [22]. In the present study, only 37.2% of patients with established ASCVD had LDL-C levels below 55 mg/dL. ASCVD-specific treatment analysis showed that 91.7% of patients received statins and 60.0% ezetimibe, with 58.6% receiving combination therapy with a statin plus ezetimibe. In contrast, only 2.8% received a PCSK9 monoclonal antibody and 1.0% inclisiran, while bempedoic acid was used in 7.7% of the ASCVD cohort. Thus, nearly two-thirds of patients with established ASCVD failed to achieve the recommended LDL-C target, while only 3.8% received PCSK9-targeted therapy and fewer than 8% received bempedoic acid. These findings highlight substantial scope for further optimisation of lipid-lowering therapy and LDL-C target attainment.

A similar gap was observed in blood-pressure management. Among patients with diagnosed hypertension, 42.6% had a clinic blood pressure ≥ 140/90 mmHg at the study visit. This proportion is consistent with previous studies conducted in Spain, which have repeatedly shown suboptimal achievement of recommended blood-pressure targets, even in cardiology or specialised care settings [23]. Together, these data underscore the continuing need to improve blood-pressure control in routine cardiology practice.

Regarding physical activity, moderate-intensity aerobic exercise for at least 150 min per week (or 75 min of vigorous activity) is recommended for adults with CVD according to the 2020 European Society of Cardiology guidelines [24]. However, in the RIESCAR cohort, 46.5% of participants reported performing at least 150 min of aerobic exercise per week, while 19.2% reported no regular physical activity. These findings reinforce the importance of promoting physical activity as part of integrated cardiovascular prevention in routine cardiology practice. Current smoking was recorded in 10.7% of RIESCAR participants, compared with 16.6% daily smoking in the Spanish population aged ≥15 years in the 2023 Spanish Health Survey [25].

Multimorbidity emerged as another defining characteristic of the RIESCAR cohort. Conditions such as chronic kidney disease, chronic obstructive pulmonary disease, sleep apnoea, and cancer survivorship frequently coexisted with CVD and further complicated clinical management. Multimorbidity inevitably increases the likelihood of polypharmacy, which was prominent in this population. While polypharmacy is often viewed negatively due to its potential impact on adherence, in cardiology it is frequently unavoidable given the complexity of contemporary guideline-directed therapy. The challenge lies not in reducing necessary medications but in optimising treatment regimens. Strategies such as polypills [26], fixed-dose combination therapies, structured medication reviews, and digital adherence tools may help mitigate the burden of complex treatment schedules.

Taken together, the findings of the RIESCAR study support the need to rethink the organisation and priorities of cardiology outpatient care. Traditional models focused primarily on CAD, arrhythmias, or valvular pathology are no longer sufficient. Instead, cardiology practice must increasingly adopt an integrated cardiometabolic approach. This includes routine assessment of central obesity, early and aggressive lipid-lowering strategies using combination therapy, proactive promotion of physical activity, use of modern cardioprotective agents such as GLP-1 receptor agonists and SGLT2 inhibitors, and close collaboration with primary care and other specialties. The objective is not only to manage cardiovascular events after they occur but to systematically modify the metabolic, structural, and inflammatory substrates that drive CVD.

### Limitations

This study has several limitations. Its cross-sectional design precludes causal inference between cardiometabolic risk factors, treatment patterns, and clinical outcomes. Participating centres volunteered after invitation and were not selected through probability sampling; therefore, although the study includes a large and geographically diverse set of general cardiology clinics, it should not be considered nationally representative. Investigators were instructed to recruit consecutive eligible patients, but no central screening log or independent monitoring system was available to verify complete adherence to consecutive recruitment. Patients older than 80 years were excluded, further limiting generalisability to the oldest patients.

Routine-care data collection resulted in variable completeness, and tests such as Lp(a), hs-CRP, urinary protein and HbA1c were obtained according to clinical indication, introducing potential indication-related selection; therefore, values observed among tested patients should not be extrapolated to the entire cohort. Blood pressure was recorded during routine care without a study-specific standardised repeated-measurement protocol, and antihypertensive treatment intensity was not specifically characterised; consequently, the observed rate of blood pressure ≥ 140/90 mmHg cannot be interpreted as evidence of therapeutic inertia or resistant hypertension. Treatment subgroup analyses identify prescribing patterns in clinically relevant populations but cannot establish formal underuse because individual contraindications, intolerance, phenotype-specific indications and reimbursement criteria were not systematically captured. The broad established-CVD composite was used to characterise cardiology case mix and should not be conflated with ASCVD, which was analysed separately. Exploratory CVD-versus-no-CVD comparisons were not adjusted for clustering by centre. Finally, self-reported or clinician-documented lifestyle variables may be subject to under-reporting, and comparisons with population surveys may be influenced by differences in age, clinical selection and variable definitions. These limitations also preclude attributing the observed cardiometabolic profile to physical inactivity or the relatively low recorded prevalence of smoking, alcohol or drug abuse to specific prevention strategies. The study was not designed to investigate sex-specific referral pathways or access to specialised cardiovascular care, and the mechanisms underlying the lower representation of women cannot be established. Diagnostic frequencies reflect the case mix of participating cardiology clinics and should not be interpreted as estimates of population prevalence or as evidence of underdiagnosis. Some variables of interest, including psychotropic medication use, uric acid levels, and prior myocarditis or infective endocarditis, were not systematically collected.

## 5. Conclusions

This study demonstrates that patients attending Spanish cardiology outpatient clinics carry a high cardiometabolic burden characterised by central obesity, diabetes, hypertension, dyslipidaemia, and established CVD. Despite frequent therapeutic intervention, important gaps remain in blood-pressure and LDL-C target attainment. Among patients with clinical conditions in which SGLT2 inhibitors or GLP-1 receptor agonists may be indicated, including diabetes, heart failure, chronic kidney disease and obesity, overall use of these therapies remained limited, particularly for GLP-1 receptor agonists, highlighting an important opportunity to improve implementation of contemporary cardiometabolic therapy in routine cardiology practice.

RIESCAR supports a holistic and multidisciplinary approach to cardiovascular prevention that systematically incorporates assessment of adiposity, optimisation of lipid and blood-pressure control, promotion of physical activity, and evidence-based cardiometabolic pharmacotherapy according to individual indications and contraindications.

## Figures and Tables

**Table 1:** Baseline characteristics and cardiovascular risk factors.

	*n*	% or Mean ± SD
**Age (years)**	2808	63.20 ± 12.84
**Women**	1032	36.75
**Employment status**	2729	
Employed	916	33.57
Unemployed	150	5.50
Retired	1488	54.53
Disability	98	3.59
Self-employed	77	2.82
**Living situation**	2672	
Living with partner	1983	74.21
Living with relatives	380	14.22
Living alone	308	11.53
Institutionalised	1	0.04
**Education level**	2594	
None	108	4.16
Primary education	927	35.74
Secondary/vocational training	954	36.78
University	605	23.32
**Hypertension**	1693	60.29
**Dyslipidaemia**	1653	58.87
**Diabetes mellitus**		
No	2093	74.54
Type 1	21	0.75
Type 2	694	24.72
**Obesity (BMI ≥ 30 kg/m^2^)**	907/2803	32.36
**Smoking status**		
Never smoker	1401	49.89
Current smoker	299	10.65
Recent former smoker	146	5.20
Former smoker	962	34.26
**Weekly aerobic exercise**		
None	539	19.20
<150 min	964	34.33
≥150 min	1305	46.47
**Alcohol or drug abuse**		
No	2639	93.98
Alcohol	158	5.63
Drugs	4	0.14
Both	7	0.25

**Table 2:** Vital signs and anthropometric measurements.

	*n*	Mean ± SD or %
Systolic blood pressure (mmHg)	2802	130.87 ± 18.67
Diastolic blood pressure (mmHg)	2802	76.04 ± 10.81
BP < 140/90 mmHg in hypertensive patients *	1693	57.36%
Heart rate (bpm)	2806	70.20 ± 12.61
Weight (kg)	2803	79.87 ± 15.90
Height (cm)	2803	168.05 ± 9.04
BMI (kg/m^2^)	2803	28.23 ± 4.92
BMI > 27 kg/m^2^	1562/2803	55.73%
BMI ≥ 30 kg/m^2^	907/2803	32.36%
Waist circumference (cm)	2776	97.74 ± 16.25
Men with waist > 102 cm	754/1760	42.84%
Women with waist > 88 cm	598/1016	58.86%
Central obesity (men > 102 cm/women > 88 cm)	1352/2776	48.70%
Central obesity with BMI < 3 0 kg/m^2^	571/2774	20.58%

Note: BMI, body mass index; BP, blood pressure. * Percentage of patients with diagnosed hypertension and blood pressure < 140/90 mmHg at the study visit.

**Table 3:** Laboratory findings.

	*n*	Mean ± SD or %	% Available
Haemoglobin (g/dL)	2770	14.40 ± 1.58	98.65
Platelets (×10^3^/mm^3^)	2769	230.80 ± 66.15	98.61
Serum creatinine (mg/dL)	2769	0.98 ± 0.54	98.61
Total cholesterol (mg/dL)	2754	167.70 ± 44.71	98.08
HDL cholesterol (mg/dL)	2738	51.28 ± 14.91	97.51
LDL cholesterol (mg/dL)	2735	92.99 ± 37.75	97.40
Non-HDL cholesterol (mg/dL)	2737	116.43 ± 40.55	97.47
Triglycerides (mg/dL)	2748	118.70 ± 68.80	97.86
Lipoprotein(a) (mg/dL)	246	58.49 ± 60.02	8.76
Apolipoprotein B (mg/dL)	137	83.13 ± 33.76	4.88
Fasting glucose (mg/dL)	2763	101.81 ± 25.12	98.40
HbA1c (%)	1828	6.07 ± 0.91	65.10
hs-CRP (mg/L)	509	1.06 ± 2.49	18.13
hs-CRP > 2 mg/L	59/509	11.59	
Urinary protein concentration (mg/dL)	538	28.71 ± 123.13	19.16
Established ASCVD patients with LDL-C < 55 mg/dL	365/981	37.21%	

Note: ASCVD, atherosclerotic cardiovascular disease; hs-CRP, high-sensitivity C-reactive protein; Lp(a), lipoprotein(a). Established ASCVD was defined as ischaemic heart disease, ischaemic stroke, or peripheral arterial disease.

**Table 4:** Main cardiovascular and metabolic therapies used.

	*n*	%
**Antiplatelet agents**	1017	36.22
Aspirin	952/1017	93.61
Ticagrelor	157/1017	15.44
Clopidogrel	124/1017	12.19
Prasugrel	36/1017	3.54
**Anticoagulants**	634	22.58
Vitamin K antagonists (VKA)	187/634	29.50
Direct oral anticoagulants (DOACs)	441/634	69.56
Other	6/634	0.95
**ACE inhibitors**	677	24.11
**Angiotensin II receptor blockers (ARBs)**	792	28.21
**Sacubitril/valsartan**	204	7.26
**Mineralocorticoid receptor antagonists**	317	11.29
**Ivabradine**	54	1.92
**Statins**	1683	59.94
Atorvastatin	817/1683	48.54
Fluvastatin	4/1683	0.24
Lovastatin	2/1683	0.12
Pitavastatin	39/1683	2.32
Pravastatin	24/1683	1.43
Rosuvastatin	581/1683	34.52
Simvastatin	215/1683	12.77
Other statins	1/1683	0.06
PCSK9 monoclonal antibodies	30	1.07
Alirocumab	19/30	63.33
Evolocumab	11/30	36.67
**Inclisiran**	10	0.36
**Ezetimibe**	788	28.06
**Bempedoic acid**	84	2.99
**SGLT2 inhibitors**	674	24.00
Empagliflozin 10 mg/day	264/674	39.17
Empagliflozin 25 mg/day	74/674	10.98
Dapagliflozin 10 mg/day	314/674	46.59
Canagliflozin 100 mg/day	12/674	1.78
Canagliflozin 300 mg/day	10/674	1.48
**GLP-1 receptor agonists**	163	5.80
Oral semaglutide	65/163	39.88
Subcutaneous semaglutide	65/163	39.88
Liraglutide	6/163	3.68
Dulaglutide	19/163	11.66
Other GLP-1 RAs	8/163	4.91
Other glucose-lowering therapies (excluding SGLT2 inhibitors and GLP-1 receptor agonists)	557	19.84
Metformin	472/557	84.74
Insulin	137/557	24.60
**Fibrates**	83	2.96
**Icosapent ethyl**	33	1.18
**Beta-blockers**	1252	44.59
**Digoxin**	37	1.32
**Calcium channel blockers**	446	15.88
**Antiarrhythmic drugs**	179	6.37
**Loop or thiazide diuretics**	746	26.57

Note: Antiplatelet-agent subtypes are not mutually exclusive because patients may receive combination antiplatelet therapy; therefore, subtype percentages can exceed 100%.

## Data Availability

The datasets generated and/or analysed during the current study are not publicly available because they contain information that could compromise participant privacy and are subject to ethical and data-protection restrictions (including GDPR). De-identified individual participant data, along with the study protocol and data dictionary, will be made available from the corresponding author upon reasonable request, subject to approval by the RIESCAR Steering Committee and completion of an appropriate data-sharing agreement.

## Supplementary Materials

The following supporting information can be downloaded at https://www.mdpi.com/article/10.3390/jcm15207916/s1, Table S1. Distribution of participants by centre; Table S2. Selected baseline characteristics according to established cardiovascular disease status, Table S3. Documented alcohol or recreational drug abuse according to established CVD status. Table S4. Use of SGLT2 inhibitors and GLP-1 receptor agonists in clinically relevant subgroups. Table S5. Lipid-lowering therapy and LDL-C in patients with and without established ASCVD.

## Abbreviations

ASCVD, atherosclerotic cardiovascular disease; BMI, body mass index; CAD, coronary artery disease; CVD, cardiovascular disease; GLP-1 RA, glucagon-like peptide-1 receptor agonist; HbA1c, glycated haemoglobin; HF, heart failure; hs-CRP, high-sensitivity C-reactive protein; LDL-C, low-density lipoprotein cholesterol; Lp(a), lipoprotein(a); LVEF, left ventricular ejection fraction; PCSK9, proprotein convertase subtilisin/kexin type 9; SGLT2, sodium–glucose cotransporter 2.
