# Supplementary material for: Clinical Characteristics, Cardiometabolic Risk Burden and Therapeutic Patterns in Patients Attending Cardiology Clinics in Spain: The RIESCAR Study
Source: J Clin Med. 2026 Oct 14;15(20):7916. doi: 10.3390/jcm15207916 (PMC13706144; doi:10.3390/jcm15207916)
Supplement: Supplementary file 1 [file jcm-15-07916-s001.zip › jcm-4561459-supplementary.pdf]

Supplementary Material – RIESCAR Study

All tables in this file are new material added in response to peer review.

Table S1. Distribution of participants by centre.

Participating centre	*n*	%
Hospital Universitario de Puerto Real	50	1.78
Hospital Universitario Virgen de las Nieves	50	1.78
Complejo Hospitalario Universitario de Jaén	50	1.78
Complejo Hospitalario de Especialidades Juan Ramón Jiménez	50	1.78
Hospital Universitario Virgen de la Victoria	99	3.53
Hospital Universitario de Cabueñes	50	1.78
Hospital General Universitario Dr. Balmis de Alicante	101	3.60
Hospital General de Elche	50	1.78
Hospital Universitario de San Juan de Alicante	50	1.78
Consorcio Hospitalario Provincial de Castellón	10	0.36
Hospital Arnau de Vilanova	50	1.78
Hospital Universitario Marqués de Valdecilla	20	0.71
Hospital General Universitario de Albacete	70	2.49
Hospital General de Valdepeñas	50	1.78
Hospital General Universitario de Toledo	37	1.32
Hospital Universitario Río Hortega	50	1.78
Hospital Clinic de Barcelona	48	1.71
Hospital de Sant Joan Despí Moisès Broggi	50	1.78
Hospital Universitari de Girona Dr. Josep Trueta	50	1.78
Hospital Universitari Joan XXIII de Tarragona	28	1.00
Hospital Universitari de Sant Joan de Reus	50	1.78
Hospital de Tortosa Verge de la Cinta	50	1.78
Hospital Comarcal de Móra d'Ebre	110	3.92
Hospital Universitario de Badajoz	50	1.78
Complexo Hospitalario Universitario A Coruña	56	1.99
Complejo Hospitalario Universitario de Ferrol	75	2.67
Hospital Universitari Son Espases	35	1.25
Hospital Universitario de Gran Canaria Dr. Negrín	70	2.49
Hospital Universitario de Canarias	60	2.14
Hospital Universitario Nuestra Señora de la Candelaria	60	2.14
Hospital Universitario Fundación Alcorcón	50	1.78
Hospital Universitario de La Princesa	50	1.78
Hospital Universitario La Paz	50	1.78
Hospital Universitario Infanta Sofía	54	1.92
Hospital Universitario Infanta Elena	50	1.78
Hospital Universitario Severo Ochoa	23	0.82
Hospital Universitario Infanta Cristina	50	1.78
Hospital Clínico Universitario Virgen de la Arrixaca	50	1.78
Hospital Universitario Basurto	50	1.78
Clínica Cardiológica Vera	100	3.56
Clínica privada Dr. Manuel Anguita Sánchez	50	1.78
Clínica privada Dr. Juan Luis Bonilla Paloma	50	1.78
Clínica de Cardiología Dr. Chopo Alcubilla	99	3.53
Hospital IMED Valencia	43	1.53
Hospital Universitario QuirónSalud Madrid	50	1.78
Hospital Universitario La Luz - QuirónSalud	50	1.78
Medicentro Leganés	100	3.56
HM Hospitales CIEC	100	3.56
Hospital Universitario de Torrejón	60	2.14
Vithas Madrid	50	1.78
**Total**	**2808**	**100.00**

Table S2. Selected baseline characteristics according to established cardiovascular disease status.

Variable	No established CVD (n=1131)	Established CVD (n=1677)	p value
Age, years	58.65 ± 14.51	66.27 ± 10.53	<0.001
Women	553 (48.89)	479 (28.56)	<0.001
Hypertension	553 (48.89)	1140 (67.98)	<0.001
Dyslipidaemia	493 (43.59)	1160 (69.17)	<0.001
Diabetes mellitus	182 (16.09)	533 (31.78)	<0.001
Never smoker	667 (58.97)	734 (43.77)	<0.001*
Current smoker	139 (12.29)	160 (9.54)	
Recent former smoker	54 (4.77)	92 (5.49)	
Former smoker	271 (23.96)	691 (41.20)	
Chronic kidney disease	48 (4.24)	231 (13.77)	<0.001
BMI, kg/m²	27.79 ± 5.20 (n=1129)	28.53 ± 4.70 (n=1674)	<0.001
Obesity (BMI ≥30 kg/m²)	336/1129 (29.76)	571/1674 (34.11)	0.016
Systolic BP, mmHg	129.67 ± 17.38 (n=1130)	131.68 ± 19.46 (n=1672)	0.044
Diastolic BP, mmHg	76.25 ± 10.98 (n=1130)	75.90 ± 10.70 (n=1672)	0.388
BP <140/90 mmHg	765/1130 (67.70)	1112/1672 (66.51)	0.510
LDL-C, mg/dL	107.65 ± 35.79 (n=1080)	83.42 ± 35.89 (n=1655)	<0.001

**Overall p value for smoking-status distribution. Comparisons are unadjusted exploratory analyses and were not adjusted for clustering by centre.*

Table S3. Documented alcohol or recreational drug abuse according to established CVD status.

Alcohol/drug abuse	No established CVD (n=1131)	Established CVD (n=1677)	p value
None	1072 (94.78)	1567 (93.44)	0.271*
Alcohol	56 (4.95)	102 (6.08)	
Drugs	2 (0.18)	2 (0.12)	
Both	1 (0.09)	6 (0.36)	

**Overall p value for the four-category distribution. Psychotropic medication use was not systematically captured in the supplied study dataset/CRF and therefore could not be analysed.*

Table S4. Use of SGLT2 inhibitors and GLP-1 receptor agonists in clinically relevant subgroups.

Clinical subgroup	Denominator	SGLT2 inhibitor, n	SGLT2 inhibitor, %	GLP-1 RA, n	GLP-1 RA, %
Diabetes mellitus	715	442	61.82	131	18.32
Heart failure	328	242	73.78	25	7.62
Chronic kidney disease	279	136	48.75	25	8.96
Obesity (BMI ≥30 kg/m²)	907	278	30.65	122	13.45
ASCVD	990	366	36.97	87	8.79
Any of diabetes/HF/CKD/obesity/ASCVD	1860	658	35.38	160	8.60

Note: These are clinically relevant subgroups, not formal treatment-eligibility denominators. Individual contraindications, intolerance, phenotype-specific eligibility, and reimbursement criteria were not captured.

Table S5. Lipid-lowering therapy and LDL-C in patients with and without established ASCVD.

Variable	No ASCVD (n=1818)	ASCVD (n=990)	p value
Statins	775 (42.63)	908 (91.72)	<0.001
Atorvastatin among statin users	335/775 (43.23)	482/908 (53.08)	
Rosuvastatin among statin users	223/775 (28.77)	358/908 (39.43)	
Simvastatin among statin users	174/775 (22.45)	41/908 (4.52)	
Other statins among statin users	43/775 (5.55)	27/908 (2.97)	
PCSK9 monoclonal antibodies	2 (0.11)	28 (2.83)	<0.001
Inclisiran	0 (0.00)	10 (1.01)	<0.001
Ezetimibe	194 (10.67)	594 (60.00)	<0.001
Statin + ezetimibe	166 (9.13)	580 (58.59)	<0.001
Bempedoic acid	8 (0.44)	76 (7.68)	<0.001
Statin + PCSK9 mAb	0 (0.00)	17 (1.72)	<0.001
Statin + inclisiran	0 (0.00)	10 (1.01)	<0.001
Ezetimibe + PCSK9 mAb	1 (0.06)	21 (2.12)	<0.001
Ezetimibe + inclisiran	0 (0.00)	7 (0.71)	0.001
LDL-C, mg/dL	106.43 ± 34.62 (n=1754)	68.96 ± 30.49 (n=981)	<0.001
LDL-C <55 mg/dL	104/1754 (5.93)	365/981 (37.21)	<0.001

Note: No patient received a PCSK9 monoclonal antibody together with inclisiran.
